# Early hemodynamic changes after transcatheter aortic valve implantation in patients with severe aortic stenosis measured by invasive pressure volume loop analysis

**DOI:** 10.1007/s12928-020-00737-4

**Published:** 2020-12-12

**Authors:** Philipp Christian Seppelt, Roberta De Rosa, Silvia Mas-Peiro, Andreas Michael Zeiher, Mariuca Vasa-Nicotera

**Affiliations:** Division of Cardiology, Department of Medicine III, University Hospital Frankfurt, Goethe University Frankfurt am Main, Frankfurt, Germany

**Keywords:** Aortic stenosis, TAVI, Hemodynamics, PV loop

## Abstract

Replacement of a stenotic aortic valve reduces immediately the ventricular to aortic gradient and is expected to improve diastolic and systolic left ventricular function over the long term. However, the hemodynamic changes immediately after valve implantation are so far poorly understood. Within this pilot study, we performed an invasive pressure volume loop analysis to describe the early hemodynamic changes after transcatheter aortic valve implantation (TAVI) with self-expandable prostheses. Invasive left ventricular pressure volume loop analysis was performed in 8 patients with aortic stenosis (mean 81.3 years) prior and immediately after transfemoral TAVI with a self-expandable valve system (St. Jude Medical Portico Valve). Parameters for global hemodynamics, afterload, contractility and the interaction of the cardiovascular system were analyzed. Left ventricular ejection fraction, (53.9% vs. 44.8%, *p* = 0.018), preload recruitable stroke work (68.5 vs. 44.8 mmHg, *p* = 0.012) and end-systolic elastance (3.55 vs. 2.17, *p* = 0.036) both marker for myocardial contractility declined significantly compared to baseline. As sign of impaired diastolic function, TAU, a preload-independent measure of isovolumic relaxation (37.3 vs. 41.8 ms, *p* = 0.018) and end-diastolic pressure (13.1 vs. 16.4 mmHg, *p* = 0.015) raised after valve implantation. Contrarily, a smaller ratio of end-systolic to arterial elastance (ventricular-arterial coupling) indicates an improvement of global cardiovascular energy efficiency (1.40 vs. 0.97 *p* = 0.036). Arterial elastance had a strong correlation with the number of conducted rapid ventricular pacings (Pearson correlation coefficient, *r* = 0.772, *p* = 0.025). Invasive left ventricular pressure volume loop analysis revealed impaired systolic and diastolic function in the early phase after TAVI with self-expandable valve for the treatment of severe aortic stenosis. Contrarily, we found indications for early improvement of global cardiovascular energy efficiency.

## Introduction

Replacement of the aortic valve is regarded as the gold standard for patients with symptomatic aortic valves stenosis. Severe aortic stenosis (AS) is primarily defined by transvalvular velocity, mean gradient and valve area according to the European and North American guidelines for the treatment of valvular heart disease. Peak velocity of 4 m/s, a mean gradient of 40 mmHg and an aortic valve area < 1.0 cm^2^ are defined as cut-off values for severe aortic stenosis [1, 2]. Aortic stenosis increases afterload and left ventricular end-diastolic pressure (LVEDP). Both entities may induce left ventricular dysfunction, an important trigger of heart failure in patients with severe aortic stenosis [3, 4]. Moreover, besides the obstruction of the left ventricular (LV) outflow tract, stiffness of the systemic arterial system plays a crucial role for afterload and contributes for LV systolic as well as diastolic dysfunction in patients with severe aortic stenosis [5, 6]. With increased afterload, the workload for the LV intensifies and the LV mass increases. Characteristically a concentric hypertrophy and LV remodeling leads to LV stiffness and eventually to increased LV afterload. The exhausted compensatory mechanism contributes finally to systolic dysfunction of the LV [7, 8]. Replacement of a stenotic aortic valve reduces immediately the ventricular to aortic gradient and is expected to improve both, the diastolic and systolic LV function over the long-term [9, 10]. Nevertheless, the immediate, intraprocedural hemodynamic changes affecting the aortic-left ventricular system after aortic valve replacement are so far poorly understood. To answer this question, we performed invasive LV pressure volume loop analysis prior and immediately after TAVI with self-expandable prothesis.

## Methods

### Patient population

Within this pilot study, eight patients with severe AS undergoing transfemoral TAVI were included in this study. The interdisciplinary heart team, consisting of an interventional cardiologist, cardiac surgeon and anaesthesiologist, made the decision for the interventions. All patients gave written informed consent for the procedure prior to intervention. Only patients older than 18 years of age, with feasible transfemoral access, absence of bleeding disorders and cardiogenic shock were included. Exclusion criteria were planned hybrid procedures, intubated and mechanically ventilated patients and urgent or emergency interventions.

Data analysis was approved by the ethics committee of the University Hospital of Frankfurt (296/16). This pilot study was not registered at a trial database, because this study did not aim to evaluate the impact of early hemodynamic changes post TAVI on longitudinal outcome measures.

### Transcatheter aortic valve implantation and PV loop measurements

PV loop measurements were conducted prior and after TAVI in a hybrid operation room. All patients received analgo-sedation and local anesthesia at the femoral puncture sites (Fentanyl 1 µg per KG body-weight and Mepivacain 1%, 40 ml). Shortly, arterial femoral access was accomplished with the use of a pre-closure device (ProGlide, Abbott Vascular, Abbott Park, Illinois, USA). For rapid ventricular pacing (RVP) a temporary pacing wire was placed in the right ventricular apex via transfemoral venous access. First, stenotic aortic valve was passed per interventionist standards and the pigtail shaped pressure volume conductance catheter (7F, CD Leycom, Zoetermeer, Netherlands) was placed into the left ventricle over a stiff guide wire (Radifocus Guidewire M Stiff, 0.025″, 260 cm, angeled distal curve, Terumo Corporation, Shibuya, Japan). PV loop analysis was performed in extrasystole free cardiac cycles. Then PV loop catheter was removed, switched for a pre-shaped stiff wire (Safari, Boston Scientific, Marlborough, USA) and a delivery sheath for the prosthetic valve was placed in the femoral vessel (all Portico Valves, St. Jude Medical Saint Paul, USA). RVP was performed as per interventionist standard either for pre-dilatation or for prosthesis post-dilatation if required. After successful valve implantation, the stiff ventricle wire was removed and another PV loop analysis was performed. Post procedure, all patients were transferred to an intermediate care unit and were monitored for at least 48 h.

### PV loop assessments

Parameters for global hemodynamics, afterload, contractility and the interactions of the cardiovascular system were measured in a steady-state for each cardiac cycle and means were used for further analysis [11]. Extrasystolic beats were excluded for analysis and data was analyzed using ConductNT software (CD Leycom, Zoetermeer, Netherlands). The mean transvalvular aortic gradient, as well as mean and systolic arterial pressure were measured invasively prior and after valve implantation by pulse wave analysis.

Reflecting global cardiac hemodynamics the following parameters were analyzed: heart rate (HR, beats/min), ejection fraction (EF,  %; EDV − ESV/EDV; EDV, end-diastolic volume, ESV, end-systolic volume), stroke volume (SV, ml; EDV-ESV), stroke volume index (SVI, ml/m^2^; SV/BSA; BSA, body surface area), stroke work (SW, mmHg × ml; defined as area of the PV loop), cardiac output (CO, l/min, HR × SV) and cardiac index (CI, l/min/m^2^; SV × HR/BSA) [11].

Myocardial contractility was assessed by end-systolic and end-diastolic volume (ESV and EDV, ml), end-systolic and end-diastolic pressure (ESP and EDP, mmHg), preload recruited stroke work (PRSW, mmHg; linear regression of stroke work with end-diastolic volume), the maximal and minimal rate of pressure change (d*P*/d*t*_max_ and d*P*/d*t*_min_, mmHg/s), the relaxation time constant Tau (ms, exponential decay of the ventricular pressure during isovolumic relaxation, defined as the time required for the LV pressure at d*P*/d*t*_min_ to be reduced by half) and the Starling contractile index (SCI, mmHg/ml  s, maximal rate of pressure change over time during isovolumetric contraction (d*P*/d*t*_max_) normalized to EDV) [11].

For determination of afterload arterial elastance (*E*_A_, mmHg/ml; ESP/SV) and valvulo-arterial impedance, an index of global left ventricular afterload (*S*_VA_, mmHg m^2^/ml; systolic arterial pressure + mean gradient)/SVI) were assessed. The interaction of left ventricle performance and the arterial load was described by end-systolic elastance (E_ES_, mmHg/ml; the slope of ESP/ESV = ESPVR, end-systolic pressure volume relationship) and end-diastolic stiffness (E_ED_, mmHg/ml; the slope of EDP/EDV = EDPVR, end-diastolic pressure volume relationship) for calculating ventricular-arterial coupling ratio (E_ES_/E_A_) [12].

Graphs displaying pre and post TAVI PV loops were plotted using Engauge digitizing software (http://digitizer.sourceforge.net/) as described before [13]. Briefly, PV loop images were converted into numerical data by turning the PV loop picture into a series of individual pressure volume data points (10 per point per limp of each curve, in total 40 data points per each PV loop). Mean for every data point was calculated and the final graph drawn using Excel (Microsoft Office 365, Microsoft, Seattle, USA).

### Data assessment and statistics

The severity of aortic valve stenosis was determined before intervention by transthoracic echocardiography (TTE), transoesophageal echocardiography and computed tomography (CT) as recommended by the European Society of Cardiology [1] [14],. Left ventricular geometry was determined by left ventricular mass index and relative wall thickness as recommended by the European Association of Cardiovascular Imaging (EACVI) and the American Society of Echocardiography (ASE) [15]. In addition to the PV-loop analysis, basic hemodynamic parameters such as heart rate, LVEDP, LVESP, systolic, mean and diastolic aortic pressure and mean aortic valve gradient were assessed by two pigtails catheters positioned in the aorta and the LV before and after TAVI. We collected a 30-day follow-up and reported adverse side events and device success according to VARC-2 [16]. The post TAVI transthoracic echocardiography was performed before discharge from the index hospitalization.

Continuous variables are shown as mean ± standard deviation and categorical data are shown as number + percentage. Hemodynamic parameters were taken as means for every patient (pre and post valve implantation) and analyzed by paired two-sided Wilcoxon–Mann–Whitney-Test for continuous variables. Subgroups (reduced left ventricular ejection fraction defined as < 40%, presences of atrial fibrillation and severe mitral valve insufficiency) were analyzed separately and differences in hemodynamic changes assessed by Wilcoxon–Mann–Whitney-Test. Non-parametric Kendall’s Tau was calculated to measure the correlation of the amount of administered contrast medium with parameters for global hemodynamics and myocardial contractility. The a priori level of statistical significance was set at *p* < 0.05 for all analyses, which were always 2-tailed and performed with SPSS, version 25 (IBM SPSS, Chicago, USA).

## Results

### Baseline characteristics and intraprocedural course

Mean age of the patients undergoing TAVI with left ventricular PV loop analysis was 81.3 years (*n* = 3 women, 37.5%) with a median STS score of 2.1% (Table 1). Cardiovascular risk factors were common, such as hypertonia (87.5%), chronic kidney disease (75%), diabetes (50%) and known coronary artery disease (50%). All patients had severe stenosis of the aortic valve with a mean aortic valve area of 0.7 cm^2^, a mean pressure gradient of 37.6 mmHg and a peak gradient of 44.7 mmHg (all values determined by transthoracic or transesophageal echocardiography prior to the intervention, Table 2). Minor aortic valve insufficiency was present in three patients (37.5%).

**Table 1:** Baseline characteristics

Age (years)	81.3 (± 5.3)
Female (*n*)	3 (37.5%)
BMI (kg/m^2^)	28.6 (± 3.2)
STS-PROM (%)^a^	2.1 (1.59–4.13)
STS-PROMM (%)^a^	11.8 (8.43–18.48)
Hypertonia (*n*)	7 (87.5%)
CKD (*n*)	6 (75%)
Diabetes (*n*)	4 (50%)
ATRIAL fibrillation (*n*)	6 (75%)
CAD (*n*)	4 (50%)
History of myocardial infarction (*n*)	2 (25%)
Previous PCI (*n*)	4 (50%)
pAVK (*n*)	2 (25%)
History of stroke (*n*)	2 (25%)
NT-proBNP (pmol/L)	0.44 (± 0.69)
Serum creatinin (mmol/L)	0.13 (± 0.05)
MDRD (ml/min/1.73 m^2^)	38.0 (± 11.7)
High sensitive Troponin-T (pg/ml)	44.5 (± 48.9)
C-reactive protein	0.94 (± 0.68)
Hemoglobin (g/L)	119 (± 23)
INR	1.27 (± 0.29)

Data are shown as mean (± standard deviation) or frequency (%)

*AI* aortic insufficiency, *AV* aortic valve, *AVA* aortic valve area, *BMI* body mass index, *CKD* chronic kidney disease, *CAD* coronary artery disease, *INR* International Normalized Ratio, *LVEDD* left ventricular end-diastolic diameter, *LVEF* left ventricular ejection fraction, *MDRD* Modification of Diet in Renal Disease formula for estimation of glomerular filtration rate, *MI* mitral valve insufficiency, *PAP* pulmonary artery pressure, *PCI* percutaneous coronary intervention, *STS-PROM and STS-PROMM* The Society of Thoracic Surgeons’ Risk model Predicting the Risk of Operative Mortality and Mortality and Morbidity, *TI* tricuspid valve insufficiency

^a^Shown as median (interquartile range)

**Table 2:** Baseline echocardiographic characteristics

LVEF (%)	44.4 (± 14.7)
LVEDD (mm)	50.9 (± 6.7)
Interventricular septum (mm)	13.6 (± 1.4)
Posterior wall thickness (mm)	12.1 (± 1.3)
LV mass (g)	270.5 (± 62.9)
LV mass index (g/m^2^)	138.6 (± 33.9)
Relative wall thickness (mm)	49 (± 1.0)
AVA (cm^2^)	0.7 (± 0.18)
AV Pmax, (mmHg)	44.7 (± 22.2)
AI	
I	3 (37.5%)
II	0 (0%)
III	0 (0%)
MI	
I	4 (50%)
II	1 (12.5%)
III	3 (37.5%)
TI	
I	6 (75%)
II	0 (0%)
III	1 (12.5%)
Systolic PAP (mmHg)	42.6 (± 11.0)

Data are shown as mean (± standard deviation) or frequency (%) and were eather assessed by transthoracic or transesophageal echocardiography at baseline. Relative wall thickness was defined as two times posterior wall thickness divided by the left ventricular (LV) end-diastolic diameter and LV mass index was defined as LV mass divided by body surface area

*AI* aortic insufficiency, *AV* aortic valve, *AVA* aortic valve area, *LV* left ventricular, *LVEDD* left ventricular end-diastolic diameter, *LVEF* left ventricular ejection fraction, *MI* mitral valve insufficiency, *PAP* pulmonary artery pressure, *PCI* percutaneous coronary intervention, *TI* tricuspid valve insufficiency

Mean ventricular mass index was 138.6 g/m^2^ and relative wall thickness 49.3 mm. In four cases LV geometry was classified as concentric left ventricular hypertrophy, in two as eccentric left ventricular hypertrophy and in two as concentric left ventricular remodeling.

In all cases self-expandable Portico Valve (Abbot Laboratories, Abbott Park, USA, mean size 27.3 mm) was implanted and a mean of one RVP per procedure either for pre-dilatation or prosthesis post-dilatation was performed (no RVP in two patients, Table 3). Significant or more than mild paravalvular leakage could be ruled out in all patients post intervention by final root angiogram and transthoracic echocardiography. Thirty-day mortality was 0% but one patient suffered from an early postoperative thrombembolic stroke and in one patient a permanent pacemaker was implanted due to third-degree atrioventricular block. Minor access site bleeding occurred in 3 patients (37.5%) but could be managed conservatively.

**Table 3:** Basic invasive hemodynamic assessment and intraprocedural data

Pre TAVI or balloon valvuloplasty	
Heart rate	59.4 (± 9.7)
Aortic pressure (mmHg) systolic/mean/diastolic)	129.6 (± 18.7)
	80.4 (± 9.4)
	54.8 (± 4.6)
Left ventricular pressure (mmHg) (systolic/LVEDP)	154 (± 19.5)
	15 (± 4.5)
AV Pmean, mmHg	25.3 (± 11.1)
AV Pmax, mmHg	28 (± 15.0)
Intraprocedural data	
Rapid ventricular pacing (*n*)	1 (± 0.77)
Contrast medium (ml)	140 (± 54.3)
Post TAVI or final balloon valvuloplasty	
Heart rate	64 (± 13.7)
Aortic pressure (mmHg) (systolic/mean/diastolic)	135 (± 21.8)
	82.6 (± 15.4)
	54.8 (± 9.6)
Left ventricular pressure (mmHg) (systolic/LVEDP)	138 (± 25.5)
	13.6 (± 4.4)
AV Pmean (mmHg)	3.75 (± 4.9)
AV Pmax (mmHg)	6 (± 5.2)

Data assessed by standard invasive, simultaneous measurement and is shown as mean (± standard deviation)

*AV* aortic valve, *LVEDP* left ventricular end-diastolic pressure, *Pmean* mean valvular gradient, *Pmax* maximal valvular gradient

### Global hemodynamics

**Fig. 1:**
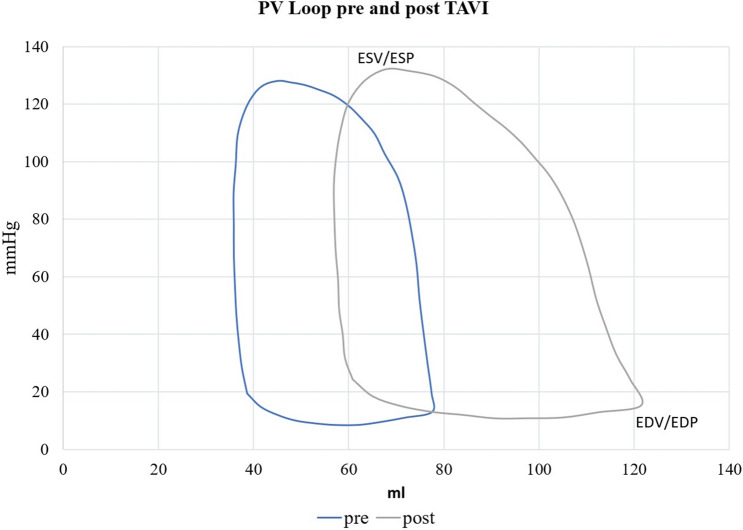
Schematic left ventricular pressure volume loops derived from the means of generated pressure volume data points of each cardiac cycle. After TAVI pressure volume loop shifts to the right and slightly upwards, indicating an increase of end-diastolic pressure (EDP) as well as end-systolic volume (ESV)

We observed a non-significant increase in SV (47 vs. 53.50 ml, *p* = 0.735) and CI (1.63 vs. 2.03 l/min, *p* = 0.31), on the other hand SW (6435 vs. 5736 mmHg ml, *p* = 0.161) and LVEF (53.9% vs. 44.8%, *p* = 0.018) decreased post prosthesis implantation (Table 4). Schematic pre and post TAVI PV loops derived from the means of each measured cardiac cycle are shown in Fig. 1.

**Table 4:** Parameters for global hemodynamics

Global hemodynamics	Pre TAVI	Post TAVI	*p* value
HR (beats/min)	64.71 (± 8.87)	74.49 (± 19.39)	0.069
EF (%)	53.88 (± 19.38)	44.75 (± 19.17)	0.018
SV (ml)	47.00 (± 18.93)	53.50 (± 37.45)	0.735
SVI (ml/m^2^)	24.20 (± 9.76)	27.64 (± 19.68)	0.735
SW (mmHg ml)	6435.13 (± 3319.02)	5736.75 (± 3985.53)	0.161
CO (l/min)	3.16 (± 1.25)	3.94 (± 2.58)	0.237
CI (l/min/m^2^)	1.63 (± 0.66)	2.03 (± 1.36)	0.31

Data assessed by PV-loop catheter and is shown as mean (± standard deviation)

*CI* cardiac index, *CO* cardiac output *EF* ejection fraction, *HR* heart rate, *SV* stroke volume, *SVI* stroke volume index, *SW* stroke work

### Myocardial contractility

Marker for myocardial contractility, such as PRSW (68.5 vs. 44.8 mmHg, *p* = 0.012) and *E*_ES_ (3.55 vs. 2.17, *p* = 0.036) both decreased significantly compared to baseline (Table 5). Relaxation time constant Tau, a preload-independent measure of isovolumic relaxation (37.3 vs. 41.8 ms, *p* = 0.018) and d*P*/d*t*_min_ (− 1168.0 vs. − 1024.5 mmHg/s, *p* = 0.036) increased and reflect a state of impaired diastolic function early after prosthesis implantation. Moreover, LVEDP (13.1 vs. 16.4 mmHg *p* = 0.015) as well as ESV (44.1 vs. 58.3%, *p* = 0.035) raised after valve implantation.

**Table 5:** Parameters for myocardial contractility

Myocardial contractility	Pre TAVI	Post TAVI	*p* value
EDV (ml)	94.00 (± 12.57)	120.13 (± 37.79)	0.094
ESV (ml)	44.13 (± 18.51)	58.25 (± 11.60)	0.035
EDP (mmHg)	13.13 (± 3.91)	16.38 (± 5.53)	0.015
ESP (mmHg)	134.88 (± 21.94)	121.75 (± 20.26)	0.176
PRSW (mmHg)	68.49 (± 32.75)	44.78 (± 20.88)	0.012
d*P*/d*t* + (mmHg/s)	1099.00 (± 300.61)	977.13 (± 288.86)	0.093
d*P*/d*t* − (mmHg/s)	− 1168.00 (± 193.39)	− 1024.50 (± 254.17)	0.036
TAU (ms)	37.25 (± 5.63)	41.75 (± 7.50)	0.018
End-systolic elastance (mmHg/ml)	3.55 (± 1.51)	2.17 (± 0.61)	0.036
SCI (mmHg/ml s)	11.85 (± 3.17)	8.88 (± 3.87)	0.069

Data assessed by PV-loop catheter and is shown as mean (± standard deviation)

*dP/dt + and dP/dt* maximum and minimum rate of pressure change, *EDP* end-diastolic pressure, *EDV* end-diastolic volume, *ESP* end-systolic pressure, *ESV* end-systolic volume, *PRSW* preload recruitable stroke work, *SCI* starling contractility index, *TAU* isovolumic relaxation constant

### Afterload and the interactions of the cardiovascular system

Arterial elastance, a measure for arterial load, remained stable after prosthesis implantation (3.61 vs. 3.67, mmHg/ml, *p* = 0.779, Table 6). The ratio of arterial to end-systolic elastance (ventricular-arterial coupling) was smaller after the procedure indicating a recovery of the cardiovascular energy efficiency (1.40 vs. 0.97 *p* = 0.036). We found a strong correlation of the absolute change in arterial elastance (difference post-minus values) with the number of conducted rapid ventricular pacings (Pearson correlation coefficient, 0.772, *p* = 0.025, mean one rapid ventricular pacing per procedure). The postprocedural course and the short-term outcome of the cohort is displayed in Table 7.

**Table 6:** Parameters for afterload and LV-afterload interactions

Afterload and LV-afterload interactions	Pre TAVI	Post TAVI	*p* value
Arterial elastance (mmHg/ml)	3.61 (± 2.51)	3.67 (± 2.87)	0.779
Valvulo-arterial impedance (mmHg m^2^/ml)	7.98 (± 5.42)	8.06 (± 6.03)	0.889
Enddiatolic stiffness (mmHg/ml)	0.14 (± 0.03)	0.15 (± 0.06)	0.575
Ventricular-arterial coupling	1.40 (± 1.04)	0.97 (± 0.69)	0.036

Data assessed by PV-loop catheter and is shown as mean (± standard deviation)

*LV* left ventricular

**Table 7:** Postprocedural course and 30-day outcome

Need for pacemaker (*n*)	1 (12.5%)
AKI with need for RRT (*n*)	0 (0%)
Minor access site bleeding (n)	3 (37.5%)
Stroke (*n*)	1 (12.5%)
Pre discharge echocardiography	
LVEF (%)	48.6 (± 20.9)
LVEDD (mm)	51.6 (± 18.9)
Interventricular septum (mm)	13.1 (± 4.8)
AV Pmean (mmHg)	7.8 (± 4.2)
AV Pmax (mmHg)	15 (± 7.2)
PVLPVL	
0	4 (50%)
II	4 (50%)
MI	
I	4 (50%)
II	4 (50%)
TI	
0	2 (25%)
I	4 (50%)
II	2 (25%)
Systolic PAP (mmHg)	38 (± 38.1)
30-day mortality (n)	0 (0%)

Data is shown as mean (± standard deviation) or frequency (%)

*AKI* acute kidney injury, *AV* aortic valve, *LVEDD* left ventricular end-diastolic diameter, *LVEF* left ventricular ejection fraction, *MI* mitral valve insufficiency, *PAP* systolic pulmonary artery pressure, *Pmean* mean valvular gradient, *Pmax* maximal valvular gradient, *PVL* paravalvular leakage, *RRR* renal replacement therapy, *TI* tricuspid valve insufficiency

### Correlation and subgroup analysis

The amount of administered contrast medium ranged from 40 to 200 ml (mean 140 ml). A correlation analysis ruled out relevant associations between administered contrast medium and parameters for global hemodynamics and myocardial contractility (Table 8).

**Table 8:** Correlations between administered contrast medium and hemodynamic parameters

Kendal Tau-b	LVEF	SVI	CI	EDV	ESV	EDP	ESP
Correlation coefficient	0.074	0.000	− 0.519	− 0.226	− 0.231	0.555	0.000
Significance (two sided)	0.802	1.000	0.079	0.448	0.444	0.081	1.000

Kendal Tau-b	SW	PRSW	TAU	d*P*/d*t* −	d*P*/d*t* +	End-systolic elastance	SCI
Correlation coefficient	0.148	0.371	0.154	0.000	0.074	0.371	0.148
Significance (two sided)	0.615	0.209	0.610	1.000	0.802	0.209	0.615

Kendal Tau B correlation analysis to assess statistical dependence between amount of administered contrast medium and parameters for global hemodynamics and myocardial contractility

*CI* cardiac index, *dP/dt + and dP/dt* maximum and minimum rate of pressure change, *EDP* end-diastolic pressure, *EDV* end-diastolic volume, *ESP* end-systolic pressure, *ESV* end-systolic volume, *PRSW LVEF* left ventricular ejection fraction, preload recruitable stroke work, *SCI* starling contractility index, *TAU* isovolumic relaxation constant

The study cohort was inhomogeneous concerning relevant comorbidities known to impact hemodynamics during TAVI, such as reduced systolic left ventricular function, prevalence of severe mitral insufficiency and atrial fibrillation. Cardiac index as well as preload recruitable stroke work declined significantly in patients with LVEF < 40% compared to patients with preserved LVEF (Table 9). Further statistical discrepancies were not found between the subgroups.

**Table 9:** Subgroup analysis

Pre to post TAVI differences	Left ventricular ejection fraction				Atrial fibrillation at presentation				Mitral valve insufficiency			
	≤ 45% (*n* = 4)		≥ 45% (*n* = 4)		No AF (*n* = 3)		AF (*n* = 5)		MI 0–II (*n* = 5)		MI III (*n* = 3)	
Global hemodynamicy												
HR (beats/min)	16 (± 20.5)		− 0.63 (± 4.27)		0.8 (± 5.09)		15.16 (± 21.2)		12.3 (± 22)		5.5 (± 8.5)	
EF (%)	− 11 (± 7)		− 6 (± 7)		− 5 (± 6)		− 12 (± 7)		− 9 (± 8)		− 9 (± 6)	
SV (ml)	20 (± 25)		− 2 (± 5)		28 (± 36)		2 (± 11)		15 (± 28)		6 (± 11)	
SVI (ml/m^2^)	− 10.81 (± 14.83)		1.24 (± 2.6)		− 14.81 (± 18.17)		− 1.18 (± 6.28)		− 8.24 (± 16.26)		− 3.04 (± 5.38)	
SW	− 555 (± 3034)		− 937 (± 391)		581 (± 3339)		− 1466 (± 1346)		− 707 (± 3054)		− 685 (± 215)	
CO (l/min)	− 1.52* (± 1.43)		− 0.46* (± 0.31)		1.55 (± 2.01)		0.32 (± 1.1)		1.35 (± 2)		− 0.18 (± 0.6)	
CI (l/min/m^2^)	− 0.76* (± 0.71)		0.22* (± 0.15)		− 0.82 (± 1.0)		− 0.14 (± 0.5)		− 0.68 (± 0.8)		0.08 (± 0.29)	
Myocardial contractility												
EDV (ml)	41 (± 40)		1 (± 16)		41 (± 57)		17 (± 25)		32 (± 47)		16 (± 20)	
ESV (ml)	21 (± 15)		3 (± 11)		13 (± 21)		15 (± 14)		17 (± 19)		10 (± 10)	
EDP (mmHg)	4 (± 6)		2 (± 1)		6 (± 7)		2 (± 2)		4 (± 6)		2 (± 1)	
ESP (mmHg)	− 18 (± 21)		− 5 (± 10)		− 11 (± 23)		− 14 (± 17)		− 14 (± 23)		− 12 (± 10)	
PRSW (mmHg)	− 32.5* (± 11)		− 9.01* (± 4.08)		− 23.52 (± 15.9)		− 23.8 (± 16.15)		− 29.4 (± 15)		− 14 (± 10)	
d*P*/d*t* + (mmHg/s)	− 133 (± 251)		− 103 (± 27)		− 234 (± 120)		− 54 (± 204)		− 154 (± 244)		− 68 (± 49)	
d*P*/d*t* − (mmHg/s)	177 (± 209)		87 (± 80)		103 (± 145)		168 (± 196)		154 (± 224)		126 (± 18)	
TAU (ms)	5 (± 7)		3 (± 3)		6 (± 10)		3 (± 2)		5 (± 8)		4 (± 2)	
End-systolic elastance (mmHg/ml)	− 0.86 (± 1.4)		1.7 (± 0.9)		− 0.85 (± 1.1)		− 0.93 (± 1.2)		− 0.83 (± 1.2)		− 1.2 (± 1)	
SCI (mmHg/ml s)	− 4.4 (± 4.61)		0.6 (± 0.98)		− 4.52 (± 4.34)		− 2.05 (± 4.03)		− 3.46 (± 5)		− 2 (± 1.9)	
Afterload and LV-afterload interactions												
Arterial elastance (mmHg/ml)	− 0.32 (± 1.05)		0.69 (± 0.5)		− 0.2 (± 1.03)		0.22 (± 1.04)		− 0.03 (± 1)		0.2 (± 0.7)	
Valvulo-arterial impedance (mmHg m^2^/ml)	− 0.56 (± 2.29)		1.15 (± 1.47)		− 0.39 (± 2.33)		0.37 (± 2.16)		0.02 (± 3)		0.2 (± 1.3)	
End-diastolic stiffness	0.1 (± 0.06)		0.03 (± 0.03)		0.03 (± 0.06)		0 (± 0.04)		0.02 (± 0.06)		0.1 (± 0.02)	
Ventricular-arterial coupling	− 0.62 (± 0.58)		− 0.12 (± 0.14)		− 0.36 (± 0.69)		− 0.48 (± 0.47)		− 0.56 (± 1)		− 0.1 (± 0.2)	

Displayed are the differences of pre and post TAVI hemodynamic data of selected subgroups assessed by PV loop analysis. Data is shown as mean (± standard deviation)

*AF* atrial fibrillation, *dP/dt + and dP/dt* maximum and minimum rate of pressure change, *EDP* end-diastolic pressure, *EDV* end-diastolic volume, *ESP* end-systolic pressure, *ESV* end-systolic volume, *PRSW* preload recruitable stroke work, *SCI* starling contractility index, *TAU* isovolumic relaxation constant *CI* cardiac index, *CO* cardiac output *EF* ejection fraction, *HR* heart rate, *SV* stroke volume, *SVI* stroke volume index, *SW* stroke work

*p < 0.05, null hypothesis rejected, Wilcoxon–Mann–Whitney-Test

## Discussion

Valve replacement in patients with severe stenosis is expected to reduce immediately the ventricular to aortic gradient in patients with preserved ejection fraction.

Beneficial long-term effects of TAVI are driven by positive remodelling of the LV, morphologically mainly due to reduction in LV wall thickness [17]. However, besides prompt gradient reduction and beneficial LV remodelling over the long term, the early hemodynamic changes immediately after valve implantation in patients with severe aortic stenosis are so far poorly understood. Our data demonstrates that besides sole, immediate gradient lowering, adverse hemodynamic effects occur in the early phase post TAVI, such as reduced myocardial contractility and impaired diastolic function. On the other hand, we found indications for improved global cardiovascular energy efficiency.

Immediately after TAVI we observed reduced LVEF, assessed invasively by PV Loop conductance catheter. Nevertheless, over the long-term TAVI is known to improve LVEF in patients with preserved as well as with reduced LVEF. The follow-up of the randomized controlled Partner A trial comparing TAVI with surgical aortic valve replacement in patients with severe aortic stenosis (PARTNER A) demonstrated, that ejection fraction improves after TAVI with most improvement occurring within the first 30 days after the procedure and independently of preoperative LVEF [18]. Markus et al. described cardiac output and cardiac index pre and post TAVI using a non-invasive whole body electrical bio-impedance monitoring system in a cohort with 52 patients. The group found cardiac output and cardiac index unchanged 6–8 h after TAVI [19]. Similar to their findings, in our study cohort, cardiac output as well as cardiac index remained stable in the early phase after TAVI.

Immediately after TAVI, we found markers for myocardial contractility to be reduced. Preload recruitable stroke work and end-systolic elastance declined significantly compared to baseline. Both are a valid marker for myocardial contractility. Moreover, preload recruitable stroke work is considered to be insensitive to preload and afterload [19, 20]. Cabaco et al. suggest that patients with aortic stenosis and preserved EF with LV hypertrophy have diminished contractile reserve, especially during increased heart rate, a condition that is given during rapid ventricular pacing [22]. However, robust data or more conclusive explanations for this phenomenon do not exist so far.

Besides reduced myocardial contractility, we found indications for impaired diastolic function early after prosthesis implantation. Relaxation time constant Tau, a preload-independent measure of isovolumic relaxation, and d*P*/d*t*_min_ increased in our cohort compared to baseline. Consequently, end-diastolic pressure as well as end-systolic volume was raised after valve implantation. This finding is surprising, as over the long-term diastolic function is expected to recover and, therefore, EDP to decline. The fundament for these favorable hemodynamic changes are a larger valve orifice and remodeling of the LV after TAVI [22, 23]. Our results suggest, that in the early phase after valve implantation, diastolic function is impaired. Our observations resemble the results of an observational study conducted by Toyota et al., who measured LVEDP during TAVI using an intracardiac catheter. The group described a mean LVEDP rise of 8.7 mmHg immediately after TAVI that was independent of paravalvular leakage or intraoperative fluid balance [25].

How do we explain our findings of impaired myocardial contractility and diastolic function in the early period after valve implantation?

Hemodynamic changes of TAVI in patients with severe aortic stenosis and LV hypertrophy are usually well-tolerated and the need for hemodynamic support is rare in clinical practice. However, one possible explanation of the early negative side-effects may be the phenomenon of temporary myocardial stunning provoked by rapid ventricular pacing [24, 25]. Myocardial stunning is characterized by a condition of postischemic mechanical dysfunction that persists after reperfusion despite the absence of irreversible injury [28].

Rapid ventricular pacing during TAVI is considered to be safe but ventricular tachycardic rate provokes demand ischemia mainly in consequence of increased myocardial oxygen demand and coronary low flow due to insufficient ventricular pump work and shortened diastole [27–30]. As a result, cardiac output drops and causes transient coronary hypoperfusion and systemic hypotension, a condition that can lead to myocardial stunning [33]. Following TAVI cardiac biomarkers sensitive for myocardial injury increase and are valid surrogates for poor long-term outcomes [32, 33]. But interestingly, the duration of rapid ventricular pacing itself seems to have no relevant correlation with periprocedural myocardial injury, defined by the elevation of ischemic markers [32].

Moreover, we found a strong correlation of the absolute change in arterial elastance with the number of conducted rapid ventricular pacings. Arterial elastance incorporates vascular load including peripheral resistance, vascular compliance and impedance as well as systolic and diastolic time intervals [36]. It is estimated by the ratio of end-systolic pressure to stroke volume, a solid marker for arterial load. More detailed, arterial elastance determines how much the aortic pressure responds to a given degree of stroke volume and allows a measure of how much the aortic pressure will rise for a given degree of cardiac ejection [37]. Arterial load itself correlates with markers for diastolic function, more exactly with relaxation time constant Tau [38]. We assume that repetitive and prolonged rapid ventricular pacing increases arterial elastance. Heart rate is an important determinant of cardiac function, and accelerated heart rate increases the ESP/SV ratio: Arterial elastance itself also incorporates information about heart rate. More exactly, about the systolic and diastolic period. Theoretically, arterial elastance is described by the three-element Windkessel model and expressed as followed: *E*_A_ = *R*_T_/[*t*_s_ + tau (1 − e^−*t*d/tau^)]; R_T_, total mean vascular resistance; *t*_s_ and *t*_d_, systolic and diastolic period; tau, diastolic pressure decay time constant [13, 36, 37]. Our analysis is consistent with the report of Freeman et al., who described the enhancement of arterial elastance during rapid ventricular pacing in an experimental dog model. Appropriate interpretation of the dynamics of arterial elastance during TAVI might help to improve periprocedural fluid and hemodynamic support management. Further studies are warranted to explain the intraprocedural changes in arterial elastance and possible implications for clinical care.

The interaction between left ventricle and arterial system can be expressed comprehensively by the ratio of arterial to end-systolic elastance, named ventricular-arterial coupling. It is recognized as an important determinant of global cardiovascular performance [38, 39]. If the ratio of arterial to end-systolic elastance is about 1, the ventricular and arterial system are considered to be optimally coupled [42]. If the ratio is greater than 1.0, stroke work significantly declines and the left ventricle works less efficiently [43]. We found a significantly lower ratio of arterial to end-systolic elastance after valve implantation compared to baseline, indicating an improvement of global cardiovascular efficiency. Lam et al. reported a smaller ratio of arterial to end-systolic elastance as a predictor for a reduction in LV mass, BNP levels and pronounced concentric left ventricular remodeling in patients with hypertension, diastolic dysfunction and preserved left ventricular function [44]. Even though the beneficial effects of valve replacement with greater valve orifice and reduced valvular gradient are not reflected in the early phase by global hemodynamics such as ejection fraction or by markers for diastolic function, improvement in ventricular-arterial coupling suggests a more sufficient interaction of the left ventricle and the arterial system.

The early phase post TAVI is often the most critical part of a TAVI procedure, sometimes requiring aggressive and prompt hemodynamic management. Our findings may give a small insight of the underlying pathophysiolocigal mechanism causing these phenomena. Our data suggest a reduction of myocardial contractility and diastolic function early after valve implantation. Especially, patients with impaired systolic or diastolic ventricular function are endangered in this part of the procedure. Our results underline the importance of a few key requirements for a successful TAVI procedure, such as avoiding unnecessary RVP, optimization of body fluid balance to reduce ventricular filling pressures and decisive vasopressor and inotropic support if needed. Besides that, PV loop analysis may help to detect and evaluate the hemodynamic relevance of a paravalvular leakage post TAVI, a condition known to be associated with worse long-term outcomes. However, larger studies with detailed hemodynamic measurements and follow-up are needed to understand the long-term effects these early hemodynamic changes may imply.

Our study has important limitations. This is a single-center study and we analyzed the data of a small and heterogeneous patient cohort, because recruitment of a larger patient sample is mainly limited by the complexity of this analysis method. Baselines left ventricular geometries were not homogeneous and due to the small cohort, a subgroup analysis to determine the impact of left ventricular geometry on hemodyanamics during TAVI was not feasible. We only performed PV loop measurements in TAVI with one self-expandable valve system, and our findings have no validity for TAVI procedures with other valve employment systems or self-expandable valves of other manufactures. Due to the small number of enrolled patients and investigators, who performed the measurements, relevant bias, such as selection bias, measurement bias, observer bias an ascertainment bias may influence our results significantly. We present observations and no statistical certainties. Therefore, a general extrapolation of our results is not valid.

## Conclusion

Invasive left ventricular pressure volume loop analysis revealed impaired systolic and diastolic function in the early phase after TAVI with self-expandable valves for the treatment of severe aortic stenosis. Left ventricular ejection fraction was impaired and end-diastolic pressure and end-systolic volume increased after valve implantation compared to baseline. Contrarily, a smaller ratio of arterial to end-systolic elastance, known as ventricular-arterial coupling, suggests an early improvement of global cardiovascular energy efficiency.

## Abbreviations

AS: Aortic stenosis
CI: Cardiac index
BSA: Body surface area
BMI: Body mass index
*E*_A_: Arterial elastance
*E*_ED_: End-diastolic stiffness
*E*_ES_: End-systolic elastance
EDP: End-diastolic pressure
EDV: End-diastolic volume
EDPVR: End-diastolic pressure volume relationship
EF: Ejection fraction
ESP: End-systolic pressure
ESPVR: End-systolic pressure volume relationship
ESV: End-systolic volume
HR: Heart rate
LV: Left ventricle
LVEDP: Left ventricular end-diastolic pressure
PV loop: Pressure volume loop
PRSW: Preload recruited stroke work
RVP: Rapid ventricular pacing
STS-PROM: The Society of Thoracic Surgeons’ Risk Model Predicting the Risk of Operative Mortality
STS-PROMM: The Society of Thoracic Surgeons’ Risk Model Predicting the Risk of Operative Mortality and Morbidity
SV: Stroke volume
SVI: Stroke volume index
*S*_va_: Valvulo-arterial impendance
SW: Stroke work

## References

[1] Baumgartner H, Falk V, Bax JJ, De Bonis M, Hamm C, Holm PJ (2018). 2017 ESC/EACTS guidelines for the management of valvular heart disease. Rev Esp Cardiol (Engl Ed)..

[2] Nishimura RA, Otto CM, Bonow RO, Carabello BA, Erwin JP, Fleisher LA (2017). AHA/ACC focused update of the 2014 AHA/ACC guideline for the management of patients with valvular heart disease: a report of the American College of Cardiology/American Heart Association Task Force on Clinical Practice Guidelines. Circulation.

[3] Hess OM, Villari B, Krayenbuehl HP. Diastolic dysfunction in aortic stenosis. Circulation. 1993;87:IV73–6. 8485837

[4] Gjertsson P, Caidahl K, Farasati M, Odén A, Bech-Hanssen O (2005). Preoperative moderate to severe diastolic dysfunction: a novel Doppler echocardiographic long-term prognostic factor in patients with severe aortic stenosis. J Thorac Cardiovasc Surg.

[5] London GM, Marchais SJ, Guerin AP, Pannier B. Arterial stiffness: pathophysiology and clinical impact. Clin Exp Hypertens. n.d.;26:689–99. 10.1081/ceh-200031982 15702623

[6] Rosca M, Magne J, Calin A, Popescu BA, Pierard LA, Lancellotti P (2011). Impact of aortic stiffness on left ventricular function and B-type natriuretic peptide release in severe aortic stenosis. Eur J Echocardiogr..

[7] Kupari M, Turto H, Lommi J (2005). Left ventricular hypertrophy in aortic valve stenosis: preventive or promotive of systolic dysfunction and heart failure?. Eur Heart J.

[8] Kimball TR, Daniels SR, Loggie JMH, Khoury P, Meyer RA (1993). Relation of left ventricular mass, preload, afterload and contractility in pediatric patients with essential hypertension. J Am Coll Cardiol.

[9] Muratori M, Fusini L, Tamborini G, Gripari P, Delgado V, Marsan NA (2016). Sustained favourable haemodynamics 1 year after TAVI: improvement in NYHA functional class related to improvement of left ventricular diastolic function. Eur Hear J Cardiovasc Imaging..

[10] Giannini C, Petronio AS, Talini E, De Carlo M, Guarracino F, Grazia M (2011). Early and late improvement of global and regional left ventricular function after transcatheter aortic valve implantation in patients with severe aortic stenosis: an echocardiographic study. Am J Cardiovasc Dis.

[11] Kerkhof PLM, Kuznetsova T, Ali R, Handly N (2018). Left ventricular volume analysis as a basic tool to describe cardiac function. Adv Physiol Educ.

[12] Sunagawa K, Maughan WL, Burkhoff D, Sagawa K (1983). Left ventricular interaction with arterial load studied in isolated canine ventricle. Am J Physiol Circ Physiol..

[13] Warriner DR, Brown AG, Varma S, Sheridan PJ, Lawford P, Hose DR (2014). Closing the loop: modelling of heart failure progression from health to end-stage using a meta-analysis of left ventricular pressure-volume loops. PLoS One..

[14] Baumgartner H, Hung J, Bermejo J, Chambers JB, Evangelista A, Griffin BP (2009). Echocardiographic assessment of valve stenosis: EAE/ASE recommendations for clinical practice. J Am Soc Echocardiogr.

[15] Marwick TH, Gillebert TC, Aurigemma G, Chirinos J, Derumeaux G, Galderisi M (2015). Recommendations on the use of echocardiography in adult hypertension: a report from the European Association of Cardiovascular Imaging (EACVI) and the American Society of Echocardiography (ASE)&dagger. J Am Soc Echocardiogr.

[16] Kappetein AP, Head SJ, Genereux P, Piazza N, van Mieghem NM, Blackstone EH (2012). Updated standardized endpoint definitions for transcatheter aortic valve implantation: the Valve Academic Research Consortium-2 consensus document. J Am Coll Cardiol.

[17] Badiani S, van Zalen J, Ramasamy A, Ozkor M, Mathur A, Kennon S (2018). 74 Left ventricular remodelling post transcatheter aortic valve implantation (TAVI) is dependent on baseline mean gradient and ejection fraction: an echocardiographic study. Heart.

[18] Elmariah S, Palacios IF, McAndrew T, Hueter I, Inglessis I, Baker JN (2013). Outcomes of transcatheter and surgical aortic valve replacement in high-risk patients with aortic stenosis and left ventricular dysfunction: results from the Placement of Aortic Transcatheter Valves (PARTNER) trial (cohort A). Circ Cardiovasc Interv..

[19] Markus B, Karatolios K, Wulle C, Pethig D, Rastan A (2019). Peri-Procedural, Non-invasive hemodynamic monitoring in TAVI-patients: potential impact on patient selection and outcome prediction. Arch Med..

[20] Chen CH, Fetics B, Nevo E, Rochitte CE, Chiou KR, Ding PYA (2001). Noninvasive single-beat determination of left ventricular end-systolic elastance in humans. J Am Coll Cardiol.

[21] Glower DD, Spratt JA, Snow ND, Kabas JS, Davis JW, Olsen CO (1985). Linearity of the Frank-Starling relationship in the intact heart: the concept of preload recruitable stroke work. Circulation.

[22] Cabaco AR, Aldalati O, Eskandari M, Silaschi M, Alcock E, Byrne J, et al. 212 Assessment of left ventricular contractile reserve in patients with severe symptomatic aortic stenosis and preserved ejection fraction. Heart. 2016;102:A140.2–A141.

[23] Costantino MF, Galderisi M, Dores E, Innelli P, Tarsia G, Di Natale M (2013). Parallel improvement of left ventricular geometry and filling pressure after transcatheter aortic valve implantation in high risk aortic stenosis: comparison with major prosthetic surgery by standard echo Doppler evaluation. Cardiovasc Ultrasound..

[24] Poulin F, Carasso S, Horlick EM, Rakowski H, Lim KD, Finn H (2014). Recovery of left ventricular mechanics after transcatheter aortic valve implantation: effects of baseline ventricular function and postprocedural aortic regurgitation. J Am Soc Echocardiogr.

[25] Toyota K, Ota T, Nagamine K, Koide Y, Nomura T, Yamanaka F (2016). Effect of transcatheter aortic valve implantation on intraoperative left ventricular end-diastolic pressure. J Anesth..

[26] Shivaraju A, Thilo C, Sawlani N, Ott I, Schunkert H, von Scheidt W (2018). Aortic valve predilatation with a small balloon, without rapid pacing, prior to transfemoral transcatheter aortic valve replacement. Biomed Res Int.

[27] Witzke C, Don CW, Cubeddu RJ, Herrero-Garibi J, Pomerantsev E, Caldera A (2010). Impact of rapid ventricular pacing during percutaneous balloon aortic valvuloplasty in patients with critical aortic stenosis: should we be using it?. Catheter Cardiovasc Interv..

[28] Marban E (1991). Myocardial stunning and hibernation. The physiology behind the colloquialisms. Circulation..

[29] Webb JG, Pasupati S, Achtem L, Thompson CR (2006). Rapid pacing to facilitate transcatheter prosthetic heart valve implantation. Catheter Cardiovasc Interv..

[30] Turer AT, Addo TA, Martin JL, Sabatine MS, Lewis GD, Gerszten RE (2011). Myocardial ischemia induced by rapid atrial pacing causes troponin T release detectable by a highly sensitive assay: insights from a coronary sinus sampling study. J Am Coll Cardiol.

[31] Hodgson JMB, John Mancini GB (1985). Relation between graded, subcritical impairments of coronary flow reserve and regional myocardial dysfunction induced by atrial pacing in dogs. J Am Coll Cardiol.

[32] Okitsu K, Iritakenishi T, Imada T, Iwasaki M, Shibata SC, Fujino Y (2017). A longer total duration of rapid ventricular pacing does not increase the risk of postprocedural myocardial injury in patients who undergo transcatheter aortic valve implantation. Heart Vessels.

[33] Fefer P, Bogdan A, Grossman Y, Berkovitch A, Brodov Y, Kuperstein R, et al. Impact of rapid ventricular pacing on outcome after transcatheter aortic valve replacement n.d. 10.1161/JAHA.118.009038 PMC6064853 29987119

[34] Barbash IM, Dvir D, Ben-Dor I, Badr S, Okubagzi P, Torguson R (2013). Prevalence and effect of myocardial injury after transcatheter aortic valve replacement. Am J Cardiol.

[35] Yong ZY, Wiegerinck EMA, Van Dijk KB, Koch KT, Vis MM, Bouma BJ (2012). Predictors and prognostic value of myocardial injury during transcatheter aortic valve implantation. Circ Cardiovasc Interv..

[36] Kelly RP, Ting CT, Yang TM, Liu CP, Maughan WL, Chang MS (1992). Effective arterial elastance as index of arterial vascular load in humans. Circulation.

[37] Freeman GL, Colston JT (1990). Role of ventriculovascular coupling in cardiac response to increased contractility in closed-chest dogs. J Clin Invest..

[38] Fukuta H, Ohte N, Wakami K, Asada K, Goto T, Mukai S (2010). Impact of arterial load on left ventricular diastolic function in patients undergoing cardiac catheterization for coronary artery disease. Circ J.

[39] Sunagawa K, Maughan WL, Sagawa K (1985). Optimal arterial resistance for the maximal stroke work studied in isolated canine left ventricle. Circ Res.

[40] Starling MR (1993). Left ventricular-arterial coupling relations in the normal human heart. Am Heart J.

[41] Little WC, Pu M (2009). Left ventricular-arterial coupling. J Am Soc Echocardiogr.

[42] De Tombe PP, Jones S, Burkhoff D, Hunter WC, Kass DA (1993). Ventricular stroke work and efficiency both remain nearly optimal despite altered vascular loading. Am J Physiol.

[43] Antonini-Canterin F, Poli S, Vriz O, Pavan D, Bello V, Nicolosi G (2013). The ventricular-arterial coupling: from basic pathophysiology to clinical application in the echocardiography laboratory. J Cardiovasc Echogr..

[44] Lam CSP, Shah AM, Borlaug BA, Cheng S, Verma A, Izzo J (2013). Effect of antihypertensive therapy on ventricular-arterial mechanics, coupling, and efficiency. Eur Heart J.

